# Correlates of sedentary behaviour and light physical activity in people living with rheumatoid arthritis: protocol for a longitudinal study

**DOI:** 10.31138/mjr.29.2.106

**Published:** 2018-06-29

**Authors:** Ciara M. O’Brien, Joan L. Duda, George D. Kitas, Jet J. C. S. Veldhuijzen van Zanten, George S. Metsios, Sally A. M. Fenton

**Affiliations:** 1 School of Sport, Exercise and Rehabilitation Sciences, University of Birmingham, Birmingham, United Kingdom,; 2 Department of Rheumatology, Russells Hall Hospital, Dudley Group NHS Foundation Trust, West Midlands, United Kingdom,; 3 Faculty of Education, Health and Wellbeing, University of Wolverhampton, Wolverhampton, United Kingdom

**Keywords:** sedentary behaviour, light physical activity, rheumatoid arthritis, accelerometry, activPAL, correlates

## Abstract

**Background:**

Sedentary behaviour (SB) is associated with adverse health outcomes in the general population. Replacing sedentary time with light intensity physical activity (LPA) has been linked with improvements in all-cause and cardiovascular disease mortality in adults. People with Rheumatoid Arthritis (RA) typically spend long periods of time sedentary, but the health consequences of ‘too much sitting’, and possible benefits of LPA, have not been fully explored in this population. Moreover, little is known regarding the determinants of these behaviours among people living with RA, and such knowledge is required for the development of effective behavioural interventions.

**Aims:**

To examine longitudinal relationships between: 1) objectively-assessed SB/LPA with health outcomes in RA, 2) hypothesised determinants of SB/LPA with objectively-assessed SB/LPA in RA.

**Methods:**

This longitudinal study will secure assessments at baseline (Time 1) and 6-month follow-up (Time 2) from RA patients. At both time points, physical assessments will be undertaken, and questionnaires administered to measure physical (e.g., percentage body fat, disease activity, physical function, pain) and psychological (e.g., depression, anxiety, vitality) health outcomes. Additional questionnaires will be administered to establish hypothesised determinants (i.e., psychosocial, individual differences, and physical environmental). Participants will wear the ActiGraph GT3X accelerometer and activPAL3^μTM^ for 7 days to objectively measure SB and LPA.

**Discussion:**

Findings will elucidate the health correlates of SB in RA, as well as the relevance of interventions targeting reductions in SB by promoting LPA. Results will also assist in identifying intervention targets (i.e., determinants), with the potential to encourage SB change in RA.

## INTRODUCTION

### Rheumatoid Arthritis

Rheumatoid Arthritis (RA) is a chronic autoimmune disease that affects approximately 0.3–1% of people worldwide.^1^ RA is characterised by high-grade local and systemic inflammation, which leads to severe joint pain, stiffness and swelling in synovial joints of the body.^2–4^ People living with RA are exposed to a 50% increase in cardiovascular risk,^5^ with heightened universal inflammation implicated in the common development of cardiovascular disease (CVD) -related morbidity and mortality in this patient group.^6,7–9^ In addition, this elevated inflammation contributes to an increased risk of poor mental health in RA.^10^

### Sedentary Behaviour

Sedentary behaviour (SB) is defined as ‘any waking behaviour characterised by an energy expenditure ≤1.5 metabolic equivalents (METS) while in a sitting, reclining, or lying posture’.^11,12^ It is distinct from physical inactivity, which refers to lack of regular engagement in moderate-to-vigorous intensity physical activity (MVPA [≥3 METS]) in accordance with physical activity recommendations (i.e., 30 minutes × 5 days per week for adults). Examples of common SBs include sitting watching television, reading a book, working at a computer and travelling in a vehicle.^13^ In the general population, SB has been consistently associated with increased inflammation, and it has been proposed that this may represent a mechanism through which SB leads to an increased risk of poor health.^14,15^ For example, prospective studies demonstrate high levels of SB to associate with worsened cardiovascular and cardiometabolic health; both of which are linked to heightened systemic inflammation.^16–25^ In addition, a recent systematic review and dose-response meta-analysis reported that total sitting time and television viewing time were associated with a greater risk of several major chronic disease outcomes, including all-cause, CVD, and cancer mortality, as well as incident diabetes.^26^ Importantly, such adverse health outcomes associated with engagement in SB have been shown to occur, despite the level of MVPA an individual engages in.^27,28^ That is, SB represents an independent risk factor for poor health.^26^

### Sedentary Behaviour and Rheumatoid Arthritis

It has been reported that people living with RA typically spend long periods of time sedentary,^3,29,30^ and recent accelerometry studies suggest people with this condition can spend up to 9 waking hours sedentary per day.^31,32^ On the basis of emerging evidence for the association between SB and inflammation, Fenton and Kitas^29^ hypothesised that high levels of SB in RA may exacerbate already elevated systemic inflammation in these patients, and contribute to the progression of RA outcomes. Akin to this proposition, Fenton et al.^31^ summarised results of non-RA studies, demonstrating the adverse links between SB with inflammation and chronic diseases with an inflammatory component (e.g., CVD, type 2 diabetes), underlining the relevance of these findings for RA. That is, as evidence for the associations between SB, inflammation and poor health continues to accumulate, it is important to evaluate the role of SB as an independent risk factor for disease outcomes in RA – a population experiencing compromised health and at high risk of comorbidity.

Whilst studies to date are yet to determine the role of SB for inflammation in RA, research has begun to examine the implications of SB for broader RA outcomes. Such investigations have employed either device-based, or self-report methods to quantify SB. For example, Khoja et al.^33^ reported positive associations between accelerometer-assessed SB with disease activity, and Greene et al.^34^ and Giles et al.^35^ showed that high levels of self-reported SB was associated with poorer physical function in RA patients. In addition, Prioreschi et al.^3^ used accelerometry to determine SB patterns in people living with RA and found an inverse association between SB and bone mass. More recently, research studies have indicated that higher levels of accelerometer-assessed SB are associated with more pain and fatigue,^36^ and a higher risk of CVD.^37^ Specifically, Fenton et al.^37^ found that total sedentary time was adversely associated with 10-year CVD risk in a sample of RA patients. Interestingly, this study was the first to examine whether the manner in which sedentary time was accumulated held implications for health outcomes in RA. Findings revealed a positive relationship between sedentary time accumulated in bouts ≥20 minutes and 10-year CVD risk.

This finding^37^ is aligned with novel prospective and experimental studies, which indicate that shorter sedentary bouts (i.e., the duration of uninterrupted sedentary periods) and more frequent sedentary breaks (i.e., interruptions in sedentary time)with light physical activity (LPA [1.6 – <3 METS]), are beneficially linked to health outcomes in non-clinical and clinical populations.^20,27,38–43^ For example, regularly breaking up SB with LPA has been associated with better cardiometabolic and cardiovascular health in adults (≥20 years)^20,38^ and older adults,^27^ and has also been linked with a reduced risk of disability among older adults.^41^ A recent systematic review and meta-analysis of experimental and observational studies also revealed that LPA (overall, and sedentary breaks) could play a role in improving adult cardiometabolic health and reducing mortality risk.^44^ In RA, recent studies have reported beneficial associations between daily LPA with CVD risk,^37^ depression and vitality.^45^

Such findings illustrating the potential health benefits of LPA are particularly relevant to people with RA, given the pain and physical dysfunction characteristic of this condition. Indeed, approaches to reduce SB by promoting LPA (‘sedentary breaks’), may be better tolerated than those targeting MVPA, the traditional focus of physical activity behaviour change interventions in this patient group. Moreover, the strong inverse correlation between SB and LPA in studies with RA patients, certainly points to the potential of behavioural interventions which aim to displace SB with LPA among this population.^33,45^

Whilst studies on this topic are beginning to emerge, a number of important limitations mark the extant literature in this field, which should be addressed in future research. First, a heavy reliance on self-report measures to assess SB and physical activity within this patient group brings issues regarding social desirability, errors in participant recall, and a tendency to underestimate levels of SB and overestimate levels of physical activity.^16,46^ Second, whilst several studies have employed accelerometers in an attempt to address the limitations of self-report, sedentariness is often incorrectly defined as activity ≤1 MET^33^ or <1 MET.^47^ As a result, SBs that require between 1–1.5 METS (e.g., sitting whilst watching television or reading) may be misclassified as LPA,^48,49^ meaning the true significance of SB for RA outcomes cannot be accurately determined. Similarly, SB is sometimes defined as ‘lack of engagement in purposeful physical activity’ (i.e., physical inactivity, not meeting MVPA guidelines), which is inconsistent with the definition used by the SB Research Network (i.e., waking behaviour ≤1.5 METS, whilst sitting/reclining/lying),^11,12^ employed almost globally across the SB literature. Finally, the majority of studies that identify associations between SB and health outcomes in RA are cross-sectional, posing a challenge when inferring a causal direction of these associations.^31^ As such, research is required to address these methodological limitations, and generate important knowledge regarding the consequences of SB in RA for inflammatory burden and related RA outcomes. It is also essential that high quality studies investigate the potential benefits of LPA participation for people living with RA, in order to elucidate the potential relevance of interventions that focus on displacing SB with LPA.

### Determinants of Sedentary Behaviour and Light Physical Activity

In order to prevent the potential adverse consequences of SB for health in RA, interventions promoting SB change should target factors that influence this behaviour (i.e., determinants). If interventions are to focus on displacing SB with engagement in LPA, they will also need to consider the determinants of LPA.

#### Psychosocial determinants and individual difference factors

*Self-efficacy.* Social Cognitive Theory (SCT)^50^ regularly serves as the theoretical basis for health behaviour change interventions.^51^ Self-efficacy (i.e., situational-specific confidence), an underlying composite of SCT, has been identified as a consistent predictor of physical activity engagement.^51^ Indeed, it has been well documented that self-efficacy is a significant determinant for the adoption and adherence of physical activity in different populations.^52–54^ Contrastingly, few studies have considered self-efficacy as a determinant for SB change, particularly in the RA population. One study by Huffman and colleagues^47^ indicated that self-efficacy for exercise was negatively and positively associated with SB and physical activity respectively in this patient group. As highlighted above, a criticism of this study is that an incorrect definition of SB as <1 MET was employed.

*Quality of motivation.* Self-Determination Theory (SDT)^55^ proposes that variability in the reasons ‘why’ a person chooses to engage (or not to engage) in a behaviour, holds important implications for levels of engagement. Specifically, SDT suggests that an individual’s motivation may vary in its degree of relative autonomy, with more autonomous reasons for engagement (e.g., fun, enjoyment, personally important) linked to an increased likelihood of adopting and persisting with engagement in a behaviour (e.g., physical activity). In contrast, more controlled reasons for participation (e.g., other people’s approval, feeling guilty) are linked to a lesser chance of sustaining behaviour. The implications of quality of motivation have been demonstrated in a considerable amount of physical activity research with different populations,^56^ including RA.^57^

SDT also postulates that humans have three basic psychological needs; namely, autonomy, competence and social relatedness. Fulfilment of these needs leads to fostering more autonomous motivation towards a behaviour, as well as benefits in mental health (e.g., vitality and well-being).^58^ SDT suggests the social environment is central to the satisfaction of these three basic needs, and holds implications for encouraging behaviour change through promoting more autonomous motivation. Specifically, the provision of autonomy support from an ‘important other’ (e.g., peer, parent, spouse, health professional) is reported to hold positive implications for need satisfaction, quality of motivation and behavioural engagement.^59–62^ Fenton et al.^45^ demonstrated perceptions of autonomy support from an ‘important other’ to be positively associated with LPA, in turn benefiting psychological health (i.e., depression and vitality), in RA patients. However, no studies have examined the role of the basic psychological needs and quality of motivation in this relationship, nor have the determinants of SB accumulation been explored in the RA population through a SDT lens. For example, it is possible that more autonomous motivation for reducing SB (e.g., identification with health benefits) is associated with lower levels of sedentariness.

#### Physical environmental determinants

The physical environment has been identified as a modifiable determinant of SB.^63^ Furthermore, the Systems of SBs framework emphasises the importance of prioritising investigation into physical environmental factors, and their relationship with SB accumulation in different populations. Distinguishing between specific factors within the physical environment (e.g., home, workplace, neighbourhood) has been stressed as important when examining the determinants of SB, in order to ensure interventions can be properly targeted. At present, research studies examining the physical environmental determinants of SB often fail to be domain-specific, which can lead to contradicting evidence.^63^

The Systems of SBs framework also depicts that elements of the physical environment may influence SB engagement in different ways, among different populations. For example, examining the determinants of SB in the workplace might not be as relevant to an older adult population as the home or neighbourhood environment might be.^63^ Indeed, aspects of the home environment, such as the number of televisions and motorised vehicles, have been positively correlated with levels of SB in adults (mean age = 57.5 years).^63^ Furthermore, aesthetic features outside of the home environment (e.g., public parks, trees) have been inversely associated with leisure-time SB in adults (mean age = 52.2 years).^65^

To date, no studies have investigated the physical environmental correlates of SB and LPA in the RA population. This patient group is highly heterogeneous, representing different ages, variability in disease activity, physical function and employment status. This underlines the need to examine physical environmental determinants across multiple domains of SB and LPA engagement (i.e., in the home, workplace and neighbourhood).

### Aims of this research

This study will address the knowledge gaps and limitations of the existing literature, in order to build an evidence base regarding the health-related correlates of SB and LPA, and hypothesised determinants of these behaviours in RA.

The aims of this study are twofold:

1. To investigate the longitudinal relationships between objectively-assessed SB patterns (i.e., overall sedentary time, sedentary bouts and sedentary breaks) and LPA, with health outcomes in people living with RA. On the basis of evidence establishing an association between SB and inflammation in non-RA cohorts, we hypothesise that SB may contribute to disease outcomes in RA via perpetuating heightened systemic inflammation. As such, our primary health outcomes are: inflammatory biomarkers (e.g., Tumour Necrosis Factor-alpha [TNF-α], Interleukin 6 [IL-6], high-sensitivity C-Reactive Protein [CRP], Erythrocyte Sedimentation Rate [ESR]), which have previously been used as primary endpoints in non-RA studies examining the association between SB, inflammation and health.^14,15^ Secondary health outcomes are: broader disease-related outcomes (e.g., disease activity, CVD risk, pain, fatigue, physical function), indices of psychological wellbeing (e.g., depression, anxiety, vitality, satisfaction with life, positive and negative affect), and quality of life.
2. To examine the longitudinal associations between hypothesised determinants (i.e., psychosocial [e.g., autonomy support], individual differences [e.g., self-efficacy] and physical environmental [e.g., home and neighbour-hood environment]) of SB and LPA (i.e., for: 1) reducing overall SB, 2) regularly breaking up SB and 3) physical activity), with objectively-assessed SB patterns (i.e., overall sedentary time, sedentary bouts and sedentary breaks) and LPA, in people living with RA.

## METHODOLOGY

### Participants and Recruitment

Participants will be recruited from Rheumatology Outpatient Clinics in a hospital in Dudley, England. Inclusion criteria will be: a clinical diagnosis of RA according to the American College of Rheumatology-European League Against Rheumatism (ACR-EULAR) Criteria and aged ≥18 years old. Exclusion criteria will be: wheelchair users and those unable to ambulate independently with the use of an assistive device.

Eligible patients will be approached about the study during Rheumatology Outpatient Clinics. A member of the research team will provide patients with information about study procedures and patients will be given the opportunity to ask the researcher any questions. Willing patients will provide informed consent to participate in the study. This study has been approved by the local National Health Service Research Ethics Committee (West Midlands – Black Country Research Ethics Committee 16/WM/0371).

### Protocol

This study will adopt a longitudinal design. Participants will be asked to visit the hospital at 2 time points; baseline (Time 1) and 6-month follow-up (Time 2). At each time point, participants will be asked to undertake 2 visits (i.e., visits 1 and 2) separated by a 7-day period. Specific protocols to be followed are described below.

#### Visit 1

Participants will visit the hospital to undertake physical assessments and complete questionnaires. At the end of Visit 1, participants will be fitted with the ActiGraph (GT3X) accelerometer and activPAL3^μTM^ posture sensor to wear for the subsequent 7 days. The researcher will give verbal and written instructions, plus a demonstration, regarding how to wear each device. Participants will also be given the Bouchard Physical Activity Record (BAR)^66^ to complete on 3 of the days during which they wear the GT3X and activPAL3^μTM^.

#### Visit 2

After 7 days, participants will re-visit the hospital to provide a fasted blood sample, and return the GT3X accelerometer, activPAL3^μTM^ posture sensor and BAR to the researcher. During this visit, they will also be asked to complete questionnaires with specific reference to their experiences of pain and fatigue over the previous 7 days.

### Measures

#### Visit 1

*Participant characteristics.* Information will be recorded pertaining to participants’ age, gender, ethnicity, marital status, education, date of diagnosis, existing chronic conditions (e.g., heart disease, diabetes, depression), current medical treatment, smoking status and living arrangements.

*Anthropometrics.* Taken in duplicate, height, weight and body composition will be measured with participants bare-foot, whilst wearing light and loose-fitting clothing. Height will be measured to the nearest 0.1cm using a stadiometer (SECA, Leicester Height Measure). Weight will be measured to the nearest 0.1kg using the Tanita Body Composition Scales (Tanita BC-418 MA P). Body Mass Index (BMI) will be calculated as weight (kg)/height^2^ (m^2^). Percent body fat and muscle mass will be measured using the Tanita Scales via bioelectrical impedance analysis.

*Resting blood pressure.* Resting blood pressure (systolic and diastolic) will be taken in duplicate with an automatic blood pressure machine (Mindray Accutorr PLUS). The blood pressure cuff will be placed over the brachial artery as standard, after the participant has rested in a supine position for 5 minutes.^36,67^

*Physical function.* Gait speed will be assessed using the 20-Metre Timed Walk Test to provide an objective measure of physical function. Participants’ time to walk from a ‘start line’ to a ‘finish line’, 20 metres apart, will be recorded using a stopwatch. The stopwatch will be started once the participant begins to walk from the ‘start line’, and will be stopped once the participant’s heel has completely crossed the ‘finish line’. Gait speed has been previously used in studies of RA to provide an objective measure of physical function (e.g., walking 15.24 metres).^68^ Research has also shown that slower gait speed is associated with RA outcomes, such as pain, fatigue and joint deformation.^69^ As such, it may also serve as a proxy for other important aspects of RA health, directly related to physical function.

*Questionnaires.* Validated questionnaires will be administered to the participant to assess self-reported RA outcomes and hypothesised determinants of SB and physical activity (see **Table 1**). Questionnaire scores will be calculated according to validated scoring instructions (e.g., mean scores will be calculated for the ‘autonomous motivation for reducing SB’ dimension in the Behavioural Regulation in Exercise Questionnaire-2).

**Table 1.** Questionnaires administered at baseline (Time 1) and 6-month follow-up (Time 2), specifically on visit 1 to the hospital

**Outcome**	**Questionnaire**	**Description**	**Example**
Pain	McGill Pain Questionnaire	Questions pertaining to the past 2 weeks 17 items - Sensory descriptors - Affective descriptors - Present pain - Average pain	For each of these words, please place a tick in onecolumn: - E.g., Throbbing - (0 = none, to 3 = severe)
Fatigue	Multidimensional Assessment of Fatigue Scale	Questions pertaining to the past 2 weeks 16 items 4 dimensions: - E.g., Degree and severity Global fatigue index	Please complete the following items based on the past 2 weeks: - E.g., To what degree have you experienced fatigue? - (1 = not at all, to 10 = a great deal)
Fatigue	Multidimensional Fatigue Inventory	Questions pertaining to the past 2 weeks 20 items 5 dimensions: - E.g., Physical fatigue	Over the past 2 weeks: - E.g., I feel fit - (1 = yes that is true, to 5 = no that is not true)
Physical function	Health Assessment Questionnaire	Questions pertaining to the past 2 weeks 8 categories: Self-reported ability to complete activities of daily living - E.g., Dressing and Grooming	Are you able to: - E.g., Dress yourself, including tying shoelaces and doing buttons? - (0 = without any difficulty, to 3 = unable to do)
Physical function	Dartmouth Coop Functional Assessment Charts	Questions pertaining to the past 2 weeks 6 items 6 dimensions: - E.g., Physical fitness	During the past 2 weeks: - E.g., what was the hardest physical activity you could do for at least 2 minutes? - (1 = very heavy, to 5 = very light)
Sleep	Pittsburgh Sleep Quality Index	Questions pertaining to the past 2 weeks 18 items Aspects of sleep - E.g., Sleep disturbances Global sleep index	In the past 2 weeks: - E.g., How often have you had trouble sleeping because you have to get up to use the bathroom? - (0 = not during the past 2 weeks, to 3 = three or more times a week)
Satisfaction with life	Satisfaction With Life Scale	Questions pertaining to the present time 5 items Satisfaction with life	I currently feel: - E.g., In most ways, my life is close to my ideal - (1 = strongly disagree, to 7 = agree)
Vitality	Subjective Vitality Scale	Questions pertaining to the past 2 weeks 6 items Vitality	Over the past 2 weeks, generally: - E.g., I have been feeling alive and vital - (1 = not at all true, to 7 = very true)
Anxiety and depression	Hospital Anxiety and Depression Scale	Questions pertaining to the past 2 weeks 14 items Depression (7 items) Anxiety (7 items)	Over the past 2 weeks: - E.g., I still enjoy the things I used to enjoy - (0 = definitely as much, to 3 = not at all)
Positive and negative affect	Positive and Negative AffectSchedule	Questions pertaining to the past 2 weeks 20 items Words describing different feelings/emotions	Over the past 2 weeks: - E.g., Interested - (1 = very slightly or not at all, to 5 = extremely)
Quality of life	World Health Organisation Quality Of Life Scale	Questions pertaining to the past 2 weeks 26 items 4 domains: - E.g., Physical health	Thinking about the past 2 weeks: - E.g., How would you rate your quality of life? - (1 = very poor, to 5 = very good)
Support from participant-identified other for physical activity, reducing sedentary behaviour and breaking up sitting time	Important Other Climate Questionnaire	6 items - Perceived autonomy support from important other regarding physical activity, reducing sedentary behaviour and breaking up sitting time	With regards to being physically active/reducing my sedentary behaviour/breaking up my sitting time: - E.g., I feel that my important other provides me with choices and options about reducing my sedentary behaviour - (1 = strongly disagree, to 7 = strongly agree)
Support from consultant for physical activity, reducing sedentary behaviour and breaking up sitting time	Health Care Climate Questionnaire	6 items - Perceived autonomy support from consultant regarding physical activity, reducing sedentary behaviour and breaking up sitting time	With regards to being physically active/reducing my sedentary behaviour/breaking up my sitting time: - E.g., I feel that my consultant understands how I see things with respect to reducing my sedentary behaviour - (1 = strongly disagree, to 7 = strongly agree)
Need satisfaction for physical activity	Psychological Need Satisfaction For Exercise Scale	Questions pertaining to the past 4 weeks 18 items - Personal experiences of physical activity	With regards to my experiences of physical activity: - E.g., I feel free to do physical activity in my own way - (1 = false, to 6 = true)
Self-efficacy for physical activity and breaking up sitting time	Self-Efficacy for Exercise Scale	9 items - Extent of self-efficacy to take part in physical activity in different situations - Extent of self-efficacy to break up sitting time in different situations	How confident would you feel taking part in physical activity (e.g., walking) 3 times per week for 20 minutes if: - E.g., You felt pain when being physically active - (1 = not confident, to 10 = very confident) How confident would you feel breaking up your sitting time every 20 minutes if: - E.g., You did not enjoy breaking up your sitting time - (1 = not confident, to 10 = very confident)
Motivation for physical activity, reducing sedentary behaviour and breaking up sitting time	Behavioural Regulation in Exercise Questionnaire-2	Questions pertaining to the past 4 weeks 19 items - Reasons for taking part in physical activity, reducing sedentary behaviour and breaking up sitting time 5 dimensions: - Intrinsic regulation (autonomous motivation) - Identified regulation (autonomous motivation) - Introjected regulation (controlled motivation) - External regulation (controlled motivation) - Amotivation	I take part in physical activity/reduce my sedentary behaviour/break up my sitting time: - E.g., Because it is fun - E.g., Because I value the benefits of doing this - E.g., Because I feel guilty when I am not doing this - E.g., Because other people say I should - E.g., But I don’t see why I should
Motivation to limit screen time	Motivation to Limit Screen Time Questionnaire	Questions pertaining to the past 4 weeks 9 items - Feelings/beliefs regarding screen-time behaviour in leisure time	I try to limit my screen-time because: - E.g., I believe too much screen time is bad for my health - (1 = not true at all, to 7 = very true)
Environmental perceptions – active travel and physical activity	Assessing Levels of Physical Activity Scale	3 sections - Home and neighbourhood environment - Walkability	About how long would it take to get from your home to the nearest businesses or facilities listed below if you walked to them? - E.g., Supermarket - (1 = 1-5 min, to 5 = more than 30 min)

*Actigraph GT3X.* The GT3X triaxial accelerometer (27g; 3.8cm × 3.7cm × 1.8cm) will be attached to an adjustable elastic belt and worn on the right hip in a vertical position.^70–74^ Participants will be asked to remove the device only for sleeping and water-based activities (e.g., swimming, bathing), and to record all replacement/removal in wear time logbooks provided. The GT3X will record accelerations in 1-second epochs, which will be converted into activity counts. These counts will then be interpreted to determine the frequency, intensity and duration of SB and physical activity.

Data collected by the GT3X will be cleaned and analysed using Actilife Software (version 6). For inclusion in analyses, participants will be required to have worn the GT3X for ≥4 days, for ≥10 hours per day, including a weekend day.^25,75–78^ Non-wear (e.g., 60 minutes of ‘zero counts’) will be defined in accordance with previous research among older adults^75,78^ and people living with RA.^45^ Time spent in SB, LPA and MVPA will be determined using cut-points that have been validated in previous research with adults (e.g., Troiano et al.),^74^ and used in studies investigating SB and physical activity in the general population,^79^ in people with rheumatic diseases^80^ and in osteoarthritis.^81^

*ActivPAL3^μTM^.* The activPAL3^μTM^ posture sensor (9g; 2.35cm × 4.3cm × 0.5cm) will be initialised using activPAL3^μTM^ software and attached by the researcher to the front of the right thigh, in a mid-anterior position, with a waterproof, adhesive Tegaderm dressing. Participants will be asked to wear the activPAL3^μTM^ for 24 hours a day to enable assessment of time spent sitting/lying, standing and stepping, as well as sit-to-stand transitions.^82–85^ activPAL3^μTM^ software will be used to download the activPAL3^μTM^ data. Sleep time will be determined using self-reported information from the wear time logbooks and BAR, in conjunction with non-wear periods identified via algorithms applied to GT3X data. Specifically, the GT3X output generated by Actilife details at which time the participant removes (e.g., at bedtime) and replaces (e.g., at waking) the accelerometer throughout the 7-day period. This will be checked against the BAR, which details (over 3 days) when the participant woke up and went to bed.

Participants will be required to have worn the activPAL3^μTM^ for ≥4 days, for ≥10 hours per day, including a weekend day, to be included in analyses.^76,86–88^ Variables derived for analyses will include: time spent sitting/lying, standing and stepping (i.e., hours per day), as well as the number of sit-to-stand transitions (i.e., sedentary breaks per day).

*Bouchard Physical Activity Record.* Participants will be asked to self-report the dominant activity undertaken every 15 minutes, over 3 days of the study week, including a weekend day (e.g., Thursday, Friday and Saturday).^66,89,90^ They will be asked to report this information in real-time, in order provide information regarding the context in which SB and physical activity were undertaken. For this study, the BAR has been adapted to include visual analogue scales, in which participants will be asked to report their average, minimum and maximum pain and fatigue on each of the 3 days. Specifically, at the end of each day, participants will be asked to mark a vertical line along a 100mm continuum from ‘no pain/fatigue’ to ‘extreme pain/fatigue’.

#### Visit 2

*Fasted blood sample.* Blood will be taken from the inside of the arm and collected in appropriate vacutainers. Standard laboratory procedures and Enzyme-Linked Immunosorbent Assays (ELISAs) will be used to measure: serum biomarkers of inflammation (i.e., TNF-α, IL-6, high-sensitivity CRP, ESR), plasma lipids (i.e., total cholesterol, high-density lipoprotein cholesterol, low-density lipoprotein cholesterol, triglycerides), plasma glucose and insulin.

*Disease activity score-28*. The number of swollen and tender joints in 28 joints of the body (i.e., hands, wrists, elbows, shoulders and knees) will be examined. Tenderness will be assessed via participants’ self-report when light pressure is applied to the joint by the researcher. The degree of swelling will be visually assessed and self-reported by the researcher. The number of swollen and tender joints will be used in conjunction with patients’ ESR and a self-reported degree of overall health ranging from 0 (very good) to 100 (very poor), to determine patients’ disease activity score-28 (DAS-28).^91^

*Questionnaires.* Validated questionnaires will be administered to the participant on visit 2 to assess RA-related pain and fatigue during the study week (see **Table 2**). Questionnaire scores will be calculated according to validated scoring instructions (e.g., pain scores from the McGill Pain Questionnaire will be calculated by summing the intensity rank values for descriptors of sensory, affective and total pain).

**Table 2.** Questionnaires administered at baseline (Time 1) and 6-month follow-up (Time 2), specifically on visit 2 to the hospital

**Outcome**	**Questionnaire**	**Description**	**Example**
Pain	McGill Pain Questionnaire	Questions pertaining to the past week 17 items - Sensory descriptors - Affective descriptors - Present pain - Average pain	For each of these words, please place a tick in one column: - E.g., Throbbing - (0 = none, to 3 = severe)
Fatigue	Multidimensional Assessment of Fatigue Scale	Questions pertaining to the past week 16 items 4 dimensions: - E.g., Degree and severity • Global fatigue index	Please complete the following items based on the past week: - E.g., To what degree have you experienced fatigue? - (1 = not at all, to 10 = a great deal)
Fatigue	Multidimensional Fatigue Inventory	Questions pertaining to the past week 20 items 5 dimensions: - E.g., Physical fatigue	Over the past week: - E.g., I feel fit - (1 = yes that is true, to 5 = no that is not true)

### Power Calculation

Power calculations were conducted with G*Power (version 3.1.9.3) using data collected from the Physical Activity in Rheumatoid Arthritis (PARA) randomised controlled trial (Trial Number: ISRCTN04121489). In the PARA study, accelerometers were utilised to measure SB, LPA and MVPA in a subsample of RA participants, and high-sensitivity CRP was measured as a biomarker of systemic inflammation. Cross-sectional accelerometer data were available for n = 61 participants. A priori power calculation from this data indicated that a sample size of n = 125 would be sufficient to detect statistically significant relationships (power = 0.80, α error of probability = .05), between daily SB and LPA with high-sensitivity CRP (a key inflammatory biomarker in RA).

To ensure the robustness of our calculations for detecting significant changes in broader RA outcomes, we also conducted power calculations for physical function, overall cardiovascular risk score and vitality. For this, cross-sectional accelerometer data, Health Assessment Questionnaire scores (physical function), QRisk-2 scores (cardiovascular risk), and vitality scores (vitality) were available for n = 61, n = 61 and n = 59 participants respectively. A priori power calculation confirmed minimum sample sizes of n = 82 (physical function), n = 14 (QRisk-2) and n = 114 (vitality), would ensure adequate statistical power (power = 0.80, α error of probability = .05) to detect the hypothesised associations.

### Statistical Analyses

SPSS (version 24) will be used to compute descriptive statistics for all measured variables. These will include information regarding participant characteristics (e.g., gender, mean age, ethnicity), health outcomes (e.g., DAS-28, BMI), determinants (e.g., quality of motivation, self-efficacy), as well as levels of SB and LPA among the RA sample. Missing value analyses will be conducted via multiple imputation^92^ or expectation maximization^93^ methods in SPSS, where missing data does not exceed 5%.

Cross-sectional associations between RA participants’ objectively-assessed SB patterns and LPA, with health outcomes (Aim 1) and proposed determinants (Aim 2) will be examined using correlation and regression analyses. Longitudinal associations from baseline (Time 1) to 6-month follow-up (Time 2) will be analysed using regression models.

*Aim 1.* For cross-sectional analyses, objectively-assessed SB patterns and LPA will be independent variables. Dependent variables will include biomarkers of inflammation, disease activity, CVD risk, pain, fatigue, physical function, depression, anxiety, vitality, satisfaction with life, positive and negative affect, and quality of life. For longitudinal analyses, regression models will examine if changes in objectively-assessed SB patterns significantly predict change in health outcomes from baseline (Time 1) to 6-month follow-up (Time 2). Health outcomes will be examined in separate regression models, but analyses will be adjusted for other factors which may influence these associations, as appropriate (e.g., disease duration, age, gender, current medication, and GT3X and activPAL3^μTM^ wear time).

*Aim 2.* For cross-sectional analyses, the determinants of SB in RA (e.g., autonomy support and self-efficacy for physical activity, reducing SB and breaking up SB) will be independent variables, and objectively-assessed SB patterns and LPA in RA will be dependent variables. For longitudinal analyses, regression models will examine if changes in hypothesised determinants of SB and LPA (e.g., autonomy support for reducing SB) significantly predicts change in objectively-assessed SB patterns and LPA from baseline (Time 1) to 6-month follow-up (Time 2). As above, all analyses will be adjusted for potential confounders (e.g., 7-day pain and fatigue [to consider the possibility of bi-directional relationships], GT3X and activPAL3^μTM^ wear time).

Subsequent analyses will use AMOS (version 24) to conduct path analyses and structural equation modelling, in order to examine multivariate relationships and hypothesised process models. For example, psychological processes (e.g., autonomy support for reducing SB) proposed to underlie objectively-assessed accumulated sedentary time and LPA engagement, in turn influencing RA disease outcomes (e.g., DAS-28) will be explored.^37,45,94,95^

## DISCUSSION

To date, there is a paucity of research on SB conducted in the RA population. The current study – investigating potential physical and psychological health consequences, as well as potential determinants, of SB and LPA in RA patients – is taking steps to address the limitations of previous studies, whilst simultaneously addressing important knowledge gaps in the field. Firstly, this study is employing two novel devices to measure SB, according to the SB Research Network definition,^11,12^ and LPA in RA patients. The present study will also provide the first longitudinal evidence regarding possible changes in health consequences associated with changes in sedentariness and LPA in this patient group. Furthermore, this longitudinal study is the first to comprehensively explore the determinants (i.e., psychosocial, individual differences and physical environmental) of SB and LPA in RA. Indeed, this study will identify modifiable factors that might influence SB and LPA in this patient group above and beyond, for example, RA-related disease activity and physical function, which may demonstrate bi-directional associations with SB and LPA. These data will elucidate targets for intervention that have the potential to support people living with RA, to reduce their time spent sedentary (e.g., by regularly breaking up their sedentary time and displacing it with LPA).

### Future Research Directions

Building on findings from this study, future research that aims to ascertain the health outcomes and determinants of SB in the RA population should be consistent in their approach. Specifically, studies should accurately define and conceptualise SB, and follow recommended protocols that utilise validated measures (subjective and objective) to measure sedentariness and its correlates in RA.^31^ Additionally, future research studies should seek to further explore the ‘sedentary-inflammation hypothesis’,^31^ which postulates cyclical relationships between SB, inflammation and adverse health outcomes in RA. Finally, interventions assessed by Randomised Controlled Trials must be developed, implemented and evaluated, to definitively test the relationships in this study in order to infer causality. Data from the present study will generate an evidence base which will inform the development of such interventions, and optimise their potential to encourage SB change and improve health outcomes among people living with RA.
